# A Special Relativistic Exploitation of the Second Law of Thermodynamics and Its Non-Relativistic Limit

**DOI:** 10.3390/e25060952

**Published:** 2023-06-17

**Authors:** Christina Papenfuss

**Affiliations:** Faculty of Engineering 2, Hochschule für Technik und Wirtschaft Berlin, Wilhelminenhofstr. 75A, 12459 Berlin, Germany; christina.papenfuss@htw-berlin.de

**Keywords:** special relativity, constitutive theory, dissipation inequality, relativistic continuum theory, relativistic balance equations, low energy limit, Liu procedure

## Abstract

A thermodynamic process is a solution of the balance equations fulfilling the second law of thermodynamics. This implies restrictions on the constitutive relations. The most general way to exploit these restrictions is the method introduced by Liu. This method is applied here, in contrast to most of the literature on relativistic thermodynamic constitutive theory, which goes back to a relativistic extension of the Thermodynamics of Irreversible Processes. In the present work, the balance equations and the entropy inequality are formulated in the special relativistic four-dimensional form for an observer with four-velocity parallel to the particle current. The restrictions on constitutive functions are exploited in the relativistic formulation. The domain of the constitutive functions, the state space, is chosen to include the particle number density, the internal energy density, the space derivatives of these quantities, and the space derivative of the material velocity for a chosen observer. The resulting restrictions on constitutive functions, as well as the resulting entropy production are investigated in the non-relativistic limit, and relativistic correction terms of the lowest order are derived. The restrictions on constitutive functions and the entropy production in the low energy limit are compared to the results of an exploitation of the non-relativistic balance equations and entropy inequality. In the next order of approximation our results are compared to the Thermodynamics of Irreversible Processes.

## 1. Introduction

The thermodynamic constitutive theory of special relativistic fluids started with the relativistic Theory of Irreversible Processes (TIP) in a paper by Eckart [1]. Relativistic TIP was generalized to mixtures of different chemical components [2,3,4] and to systems with polarization and magnetization [5]. For a review of the problems and possible solutions of special- and general-relativistic TIP see [6] and for an overview over the literature on relativistic TIP [7,8].

The most general method to exploit the implications of the Second Law of Thermodynamics on constitutive functions is the method of Liu [9]. A thermodynamic process is a solution of the balance equations fulfilling the dissipation inequality. This imposes constraints on the constitutive functions [10]. The result of this exploitation depends on the set of variables the constitutive functions are defined on, the state space. The choice of the state space is always the first step in the procedure. Here, the state space is a first order gradient one.

In relativistic continuum theory, the method of Liu is mostly applied in the context of Extended Thermodynamics [11], which is often motivated by kinetic theory, see f.i. [12]. Relativistic kinetic theory is based on the Boltzmann–Chernikov equation for the statistical distribution function, the balance equations being the moment equations. They have been derived for monatomic as well as polyatomic gases, see f.i. [12,13]. In this approach, there is always the necessity of a closure relation, for example, maximum entropy closure. The fact that the Boltzmann–Chernikov equation is valid for dilute gases only is a limitation of this kinetic theory. The kinetic theory of dense gases or even liquids requires two-particle or many-particle distribution functions and assumptions about particle-particle interactions. In non-relativistic theory it leads to the BBGKY hierarchy. Instead of particle–particle interactions, continuum theory introduces constitutive functions.

In [14], the Liu procedure with the energy–momentum balance as a constraint led to the conclusion that in the relativistic case it is not possible to have local Lagrange–Farkas multipliers, a local entropy, and no additional term in the entropy flux (the assumptions of relativistic TIP) simultaneously. This observation coincides with our observation here with respect to the relativistic correction terms (the order $\frac{1}{c^{5}}$).

We will apply here method of Liu to exploit the implications of the Second Law of Thermodynamics on constitutive functions. In the non-relativistic context this exploitation of the dissipation inequality with a first order gradient state space leads exactly to the assumptions (local equilibrium and the special form of the entropy flux) and results (the entropy production) of TIP. This non-relativistic example is summarized in Section 2. In Section 3, the balance equations of special relativistic continuum theory are formulated in Lorentz covariant form and in the decomposition for an observer with four-velocity ***u*** with Eckarts choice of the four-velocity. To prepare the low energy approximation, the kinematic quantities are expanded in a series of $\frac{v}{c}$. In Section 4, the Second Law of Thermodynamics is exploited with the balance equations for an observer as constraints. In lowest non-trivial order $\frac{1}{c^{3}}$ the non-relativistic TIP is recovered. In the order of approximation $\frac{1}{c^{5}}$ it cannot be concluded, in general, that the entropy flux is heat flux divided by temperature; however, there may be an extra entropy flux. Local equilibrium η(e,n) still implies that the extra entropy flux vanishes. Finally, the entropy production with relativistic correction terms is derived.

Throughout this paper, we will deal with a one-component system (no mixture of chemical components), which is a simple fluid (no internal angular momentum and no antisymmetric part of the stress tensor). We will restrict ourselves to special relativity with the convention for the Minkowski tensor diag(−1,1,1,1). For the indices of four-vectors and tensors, we will use capital Latin letters to denote components from 0 to 3 (i.e., time and space components) and Greek letters to denote components from 1 to 3 (i.e., space components only).

## 2. Non-Relativistic Continuum Thermodynamics: Example of a Simple, Viscous, Heat Conducting Fluid

### 2.1. Balance Equations

We have the following set of balance equations for the wanted fields of mass density ϱ, material velocity ***v***, and specific internal energy *e*:

(1) $$\begin{array}{c} \mathrm{Mass}:\frac{d\varrho }{dt}+\varrho \nabla \cdot v=0\\ \mathrm{Momentum}:\varrho \frac{dv}{dt}-\nabla \cdot t-\varrho f=0\\ \mathrm{Internal}\;\mathrm{energy}:\varrho \frac{de}{dt}+\nabla \cdot q-t:\nabla v=0 \end{array}$$

with a symmetric stress tensor ***t***, because the fluid is not micro-polar. ***q*** is the heat flux and $\frac{d}{dt}=\frac{\partial }{\partial t}+v\cdot \nabla$ denotes the material time derivative.

In addition, we have the balance of entropy with a non-negative production σ

(2) $$\begin{array}{c} \mathrm{Entropy}\;\mathrm{inequality}\;\varrho \frac{d\eta }{dt}+\nabla \cdot \phi =\sigma \geq 0 \end{array}$$

with specific entropy η and entropy flux ϕ.

Stress tensor, heat flux, entropy, and entropy flux are constitutive quantities. Constitutive functions depend on the state space, here a first order gradient one

(3) ‒Z={ϱ,e,∇ϱ,∇e,∇v}

### 2.2. Exploitation of the Dissipation Inequality

The space and time derivatives of constitutive functions in the balance equations are carried according to the chain rule. The balance equations are considered as constraints, and the following inequality is exploited:

(4) $$\begin{array}{c} Balance\;of\;Entropy+\lambda^{\varrho }\left(Balance\;of\;mass\right)+\lambda^{e}\left(Balance\;of\;internal\;energy\right)+\\ +\lambda^{v}\left(Balance\;of\;momentum\right)\geq 0 \end{array}$$

The prefactors in front of the higher derivatives lead to the Liu equations:

(5) $$\begin{array}{ccc} \dot{\varrho }:\;\lambda^{\varrho } & = & -\varrho \frac{\partial \eta }{\partial \varrho }, \end{array}$$

(6) $$\begin{array}{ccc} \dot{e}:\;\varrho \lambda^{e} & = & -\varrho \frac{\partial \eta }{\partial e}, \end{array}$$

(7) $$\begin{array}{ccc} \dot{v}:\;\varrho \lambda^{v} & = & 0, \end{array}$$

(8) $$\begin{array}{ccc} \left(\nabla \varrho \right){}^{˙}:\;0 & = & \varrho \frac{\partial \eta }{\partial \nabla \varrho }, \end{array}$$

(9) $$\begin{array}{ccc} \left(\nabla e\right){}^{˙}:\;0 & = & \varrho \frac{\partial \eta }{\partial \nabla e}, \end{array}$$

(10) $$\begin{array}{ccc} \left(\nabla v\right){}^{˙}:\;0 & = & \varrho \frac{\partial \eta }{\partial (\nabla v)}, \end{array}$$

(11) $$\begin{array}{ccc} \nabla \nabla \varrho :\;-\lambda^{e}\frac{\partial q}{\partial \nabla \varrho } & = & \frac{\partial \Phi }{\partial \nabla \varrho }, \end{array}$$

(12) $$\begin{array}{ccc} \nabla \nabla e:\;-\lambda^{e}\frac{\partial q}{\partial \nabla e} & = & \frac{\partial \Phi }{\partial \nabla e}, \end{array}$$

(13) $$\begin{array}{ccc} \nabla \nabla v:\;-\lambda^{e}\frac{\partial q}{\partial \nabla v} & = & \frac{\partial \Phi }{\partial \nabla v}. \end{array}$$

From Equations (8)–(10), it follows that η=η(ϱ,e), i.e., local equilibrium.

Consequently,

(14) $$\lambda^{\varrho }=\lambda^{\varrho }\left(\varrho ,e\right),\;\lambda^{e}=\lambda^{e}\left(\varrho ,e\right)=-\frac{1}{T}.$$

Temperature *T* has been introduced by the equilibrium relation $\frac{1}{T}=\frac{\partial \eta }{\partial e}=-\Lambda^{e}$.

The extra entropy flux

$$\Phi +\lambda^{e}q=k\left(\varrho ,e\right)=0$$

in an isotropic material, because it is a function of scalar variables only. We have the classical relation of TIP $\Phi =\frac{q}{T}$.

With the definition

(15) $$t=-p\left(\varrho ,e\right)1+t^{\mathrm{dyn}}$$

the entropy production reads

(16) $$\sigma =\underset{viscous\;flow}{\underset{︸}{\frac{1}{T}t^{\mathrm{dyn}}:{\left(\nabla v\right)}^{sym}}}+\underset{heat\;conduction}{\underset{︸}{q\cdot \nabla \frac{1}{T}}}$$

## 3. Relativistic Balance Equations

The balance equations of particle number (replacing the balance of mass of the classical theory), energy-momentum, and entropy are formulated in terms of the four-vector of particle number $N^{A}$, the symmetric energy-momentum tensor $T^{BA}$ and the four-vector of entropy $S^{A}$. For an observer at rest (i.e., in the non-relativistic limit) the 0-component of this entropy-four-vector is the entropy density. For this observer, the 1,2,3-components of $S^{A}$ are the components of the entropy flux.

The conservation of particle number, conservation of energy, and momentum are formulated as

(17) $$\begin{array}{ccc} Particle\;number: & & N_{\;,A}^{A}=0\;A=0,1,2,3 \end{array}$$

(18) $$\begin{array}{ccc} Energy\text{-}momentum: & & T_{\;,A}^{BA}=0\;A,B=0,1,2,3. \end{array}$$

For energy-momentum it is supposed, that there is no supply. The

(19) $$\begin{array}{c} Entropy\;inequality\;S_{,A}^{A}=\sigma \geq 0 \end{array}$$

expresses the Second Law of Thermodynamics. The entropy production σ is a Minkowski-scalar, i.e., observer independent.

### Decomposition for an Observer

The four-velocity of an observer is introduced parallel to the particle current $N^{A}=nu^{A}$ (Eckarts choice of the four-velocity ***u***) [1] with particle density *n*. This choice is motivated by the analogy to the classical theory, where the material velocity is defined by streamlines of particles.

For an observer with four-velocity ***u***, we have the decomposition

(20) $$\begin{array}{c} N^{A}=nu^{A} \end{array}$$

(21) $$\begin{array}{c} T^{AB}=\frac{1}{c^{2}}\left(Eu^{A}u^{B}+q^{B}u^{A}+q^{A}u^{B}\right)-t^{AB} \end{array}$$

(22) $$\begin{array}{c} S^{A}=n\eta u^{A}+\Phi^{A} \end{array}$$

with *E*: total energy density, ***q***: heat flux, ***t***: stress tensor, nη: entropy density, (η: entropy per particle), and Φ: entropy flux. Details about the relativistic balance equations and the decomposition for an observer can be found, for example, in [2,15].

With the aid of the projector $h_{B}^{C}=\delta_{B}^{C}+\frac{1}{c^{2}}u^{C}u_{B}$, the energy–momentum balance is decomposed into

(23) $$\begin{array}{ccc} u_{B}T_{\;,A}^{BA} & = & 0\;balance\;of\;energy \end{array}$$

(24) $$\begin{array}{ccc} h_{B}^{C}T_{\;,A}^{BA} & = & 0\;balance\;of\;momentum \end{array}$$

with A,B,C=0,1,2,3. The components $t^{0\alpha }$ and $t^{00}$ (α=1,2,3) are eliminated by use of the relations $t^{AB}u_{B}=t^{A0}u_{0}+t^{A\beta }u_{\beta }=0$. Analogously, we have $q^{A}u_{A}=0$, i.e., $q^{0}u_{0}=-q^{\alpha }u_{\alpha }$.

Explicitly the set of balance equations for an observer with four-velocity

(25) $$u^{A}=\gamma \left(c,v^{\alpha }\right),\;\gamma :=\frac{1}{\sqrt{1-\frac{v^{2}}{c^{2}}}}$$

reads in three-dimensional notation (Greek indices run from 1,…,3):

Balance of particle number

(26) $$\begin{array}{c} \frac{1}{c}\left(\frac{dn}{dt}+n\partial_{\mu }v^{\mu }\right)+\frac{1}{c^{3}}\gamma^{2}na_{\mu }v^{\mu }=0, \end{array}$$

where $a_{\mu }=\frac{dv_{\mu }}{dt}$.

From total energy density the ‘rest-mass-energy’ $m_{0}nc^{2}$ with rest mass per particle $m_{0}$ is subtracted

(27) $$ne=E-m_{0}nc^{2}$$

*e*: internal energy per particle. By the aid of the balance of particle number (26), the balance of energy (23) results in the balance of internal energy

(28) $$\begin{array}{c} \frac{1}{c^{3}}\left(\frac{\partial ne}{\partial t}+\partial_{\nu }\left(nev^{\nu }\right)+\frac{1}{\gamma }\partial_{\nu }q^{\nu }-t^{\mu \nu }\partial_{\mu }v_{\nu }\right)+\\ +\frac{1}{c^{5}}\left(\gamma^{2}nev^{\nu }a_{\nu }+\gamma q^{\nu }a_{\nu }+\frac{1}{\gamma }\frac{\partial }{\partial t}\left(q^{\nu }v_{\nu }\right)-t^{\mu \nu }v_{\nu }\frac{\partial }{\partial t}v_{\mu }\right)=0 \end{array}$$

From the balance of momentum (24) we obtain

(29) $$\begin{array}{c} \frac{1}{c^{2}}\left(na^{\mu }-\partial_{\nu }t^{\mu \nu }\right)+\frac{1}{c^{4}}\left(nea^{\mu }+\gamma^{2}na^{\nu }v_{\nu }v^{\mu }-\frac{1}{\gamma^{2}}\frac{\partial }{\partial t}\left(v_{\nu }t^{\mu \nu }\right)+\right.\\ \left.+t^{\alpha \beta }v^{\mu }\partial_{\beta }v_{\alpha }+\frac{1}{\gamma }\frac{\partial }{\partial t}q^{\mu }+\frac{1}{\gamma }q_{\nu }\partial^{\mu }v^{\nu }\right)+O\left(\frac{1}{c^{6}}\right)=0 \end{array}$$

For γ, $\gamma^{2}$, and $\frac{1}{\gamma }$, the following series of expansions are inserted:

(30) $$\begin{array}{c} \gamma =\frac{1}{\sqrt{1-\frac{v^{2}}{c^{2}}}}=1+\frac{v^{2}}{2c^{2}}+O\left(\frac{1}{c^{4}}\right) \end{array}$$

(31) $$\begin{array}{c} \gamma^{2}=\frac{1}{1-\frac{v^{2}}{c^{2}}}=1+\frac{v^{2}}{c^{2}}+O\left(\frac{1}{c^{4}}\right) \end{array}$$

(32) $$\begin{array}{c} \frac{1}{\gamma }=\sqrt{1-\frac{v^{2}}{c^{2}}}=1-\frac{v^{2}}{2c^{2}}+O\left(\frac{1}{c^{4}}\right) \end{array}$$

Finally, the balance equations are written in a more compact form in terms of the specific quantities (quantities per particle) and the material time derivative $\frac{d}{dt}$ (see the analogy in the case of the non-relativistic balance equations). All partial time derivatives are replaced by material time derivatives by $\frac{\partial }{\partial t}=\frac{d}{dt}-v^{\mu }\partial_{\mu }$. The final form of the balance equations up to order $\frac{1}{c^{5}}$ reads

**Particle number:**

(33) $$\begin{array}{c} \frac{1}{c}\left(\frac{dn}{dt}+n\partial_{\mu }v^{\mu }\right)+\frac{1}{c^{3}}\left(1-\frac{v^{2}}{c^{2}}\right)na_{\mu }v^{\mu }=0 \end{array}$$

**Internal energy:**

(34) $$\begin{array}{c} \frac{1}{c^{3}}\left(n\frac{de}{dt}+\left(1-\frac{1}{2}\frac{v^{2}}{c^{2}}\right)\partial_{\mu }q^{\mu }-t^{\mu \nu }\partial_{\mu }v_{\nu }\right)+\\ +\frac{1}{c^{5}}\left(v_{\mu }\frac{dq^{\mu }}{dt}-v_{\mu }v^{\alpha }\partial_{\alpha }q^{\mu }+2q^{\mu }a_{\mu }-q^{\mu }v_{\nu }\partial_{\mu }v^{\nu }+t^{\mu \nu }v_{\mu }a_{\nu }+t^{\mu \nu }v_{\mu }v^{\alpha }\partial_{\alpha }v_{\nu }\right)=0 \end{array}$$

**Momentum:**

(35) $$\begin{array}{c} \frac{1}{c^{2}}\left(na^{\mu }-\partial_{\nu }t^{\mu \nu }\right)+\frac{1}{c^{4}}\left(na^{\nu }v_{\nu }v^{\mu }+nea^{\mu }+v^{2}\partial_{\nu }t^{\mu \nu }-a_{\nu }t^{\mu \nu }-v_{\nu }\frac{dt^{\mu \nu }}{dt}+\right.\\ \left.+v_{\nu }v^{\alpha }\partial_{\alpha }t^{\mu \nu }+v^{\alpha }t^{\mu \nu }\partial_{\alpha }v_{\nu }-v^{\mu }t^{\beta \gamma }\partial_{\beta }v_{\gamma }+\frac{dq^{\mu }}{dt}+q_{\nu }\partial^{\mu }v^{\nu }+q_{\mu }\partial^{\nu }v^{\nu }\right)=0 \end{array}$$

**Entropy**

(36) $$\begin{array}{c} \frac{1}{c^{3}}\left(n\frac{d\eta }{dt}+\partial_{\mu }\Phi^{\mu }\right)+\\ +\frac{1}{c^{5}}\left(-v^{2}n\frac{d\eta }{dt}+\Phi^{\mu }a_{\mu }+v_{\mu }\frac{d\Phi^{\mu }}{dt}-v^{\mu }\Phi^{\alpha }\partial_{\mu }v_{\alpha }-v^{\mu }v_{\alpha }\partial_{\mu }\Phi^{\alpha }\right)=\frac{1}{c^{3}}\sigma \geq 0 \end{array}$$

The non-relativistic balance equations (see Section 2.1) are recovered in order $\frac{1}{c^{3}}$ in the case that $a_{\mu }v^{\mu }=0$, as was noticed in [16].

## 4. Exploitation of the Second Law of Thermodynamics

The state space is chosen in analogy to the non-relativistic example

(37) $$‒\;Z=\{n,e,\partial_{\mu }n,\partial_{\mu }e,\partial_{\mu }v^{\nu }\}$$

The balance equations with multipliers are added to the balance of entropy:

(38) $$\begin{array}{c} balance\;of\;entropy\;(36)+\Lambda^{n}\left(balance\;of\;particle\;number\;(33)\right)+\\ +\Lambda^{e}\left(balance\;of\;internal\;energy\;(34)\right)+\Lambda^{v}\cdot \left(balance\;of\;momentum\;(24)\right)\geq 0. \end{array}$$

The resulting expression is linear in the higher derivatives, not included in the state space. The factors in front of the higher derivatives lead to the following restrictions on constitutive functions:

$$\begin{array}{ccc} \frac{dn}{dt} & : & 0=\frac{1}{c}\Lambda^{n}+\frac{1}{c^{3}}n\frac{\partial \eta }{\partial n}\left(1-\frac{v^{2}}{c^{2}}\right)+\frac{1}{c^{5}}v_{\alpha }\left(\frac{\partial \Phi^{\alpha }}{\partial n}+\Lambda^{e}\frac{\partial q^{\alpha }}{\partial n}\right)+ \end{array}$$

(39) $$\begin{array}{ccc} & \; & +\frac{1}{c^{4}}\Lambda_{\mu }^{v}\left(-v_{\nu }\frac{\partial t^{\mu \nu }}{\partial n}+\frac{\partial q^{\mu }}{\partial n}\right) \end{array}$$

(40) $$\begin{array}{ccc} O(\frac{1}{c^{3}}) & : & c^{2}\Lambda^{n}=-n\frac{\partial \eta }{\partial n}\\ \frac{de}{dt} & : & 0=\frac{1}{c^{3}}n\left(\frac{\partial \eta }{\partial e}\left(1-\frac{v^{2}}{c^{2}}\right)+\Lambda^{e}\right)+\frac{1}{c^{5}}v_{\alpha }\left(\frac{\partial \Phi^{\alpha }}{\partial e}+\Lambda^{e}\frac{\partial q^{\alpha }}{\partial e}\right)+ \end{array}$$

(41) $$\begin{array}{ccc} & \; & +\frac{1}{c^{4}}\Lambda_{\mu }^{v}\left(-v_{\nu }\frac{\partial t^{\mu \nu }}{\partial e}+\frac{\partial q^{\mu }}{\partial e}\right) \end{array}$$

(42) $$\begin{array}{ccc} O(\frac{1}{c^{3}}) & : & \Lambda^{e}=-n\frac{\partial \eta }{\partial e}\\ \frac{d\partial_{\mu }n}{dt} & : & 0=\frac{1}{c^{3}}n\frac{\partial \eta }{\partial \partial_{\mu }n}\left(1-\frac{v^{2}}{c^{2}}\right)+\frac{1}{c^{5}}v_{\alpha }\left(\frac{\partial \Phi^{\alpha }}{\partial \partial_{\mu }n}+\Lambda^{e}\frac{\partial q^{\alpha }}{\partial \partial_{\mu }n}\right)+ \end{array}$$

(43) $$\begin{array}{ccc} & \; & +\frac{1}{c^{4}}\Lambda_{\alpha }^{v}\left(-v_{\nu }\frac{\partial t^{\alpha \nu }}{\partial \partial_{\mu }n}+\frac{\partial q^{\alpha }}{\partial \partial_{\mu }n}\right) \end{array}$$

(44) $$\begin{array}{ccc} O(\frac{1}{c^{3}}) & : & \frac{\partial \eta }{\partial \partial_{\mu }n}=0\\ \frac{d\partial_{\mu }e}{dt} & : & 0=\frac{1}{c^{3}}n\frac{\partial \eta }{\partial \partial_{\mu }e}\left(1-\frac{v^{2}}{c^{2}}\right)+\frac{1}{c^{5}}v_{\alpha }\left(\frac{\partial \Phi^{\alpha }}{\partial \partial_{\mu }e}+\Lambda^{e}\frac{\partial q^{\alpha }}{\partial \partial_{\mu }e}\right)+ \end{array}$$

(45) $$\begin{array}{ccc} & \; & +\frac{1}{c^{4}}\Lambda_{\alpha }^{v}\left(-v_{\nu }\frac{\partial t^{\alpha \nu }}{\partial \partial_{\mu }e}+\frac{\partial q^{\alpha }}{\partial \partial_{\mu }e}\right) \end{array}$$

(46) $$\begin{array}{ccc} O(\frac{1}{c^{3}}) & : & \frac{\partial \eta }{\partial \partial_{\mu }e}=0\\ \frac{d\partial_{\mu }v^{\beta }}{dt} & : & 0=\frac{1}{c^{3}}n\frac{\partial \eta }{\partial \partial_{\mu }v^{\beta }}\left(1-\frac{v^{2}}{c^{2}}\right)+\frac{1}{c^{5}}v_{\alpha }\left(\frac{\partial \Phi^{\alpha }}{\partial \partial_{\mu }v^{\beta }}+\Lambda^{e}\frac{\partial q^{\alpha }}{\partial \partial_{\mu }v^{\beta }}\right)+ \end{array}$$

(47) $$\begin{array}{ccc} & \; & +\frac{1}{c^{4}}\Lambda_{\alpha }^{v}\left(-v_{\nu }\frac{\partial t^{\alpha \nu }}{\partial \partial_{\mu }v^{\beta }}+\frac{\partial q^{\alpha }}{\partial \partial_{\mu }v^{\beta }}\right) \end{array}$$

(48) $$\begin{array}{ccc} O(\frac{1}{c^{3}}) & : & \frac{\partial \eta }{\partial \partial_{\mu }v^{\beta }}=0\\ a^{\mu } & : & 0=\Lambda_{\mu }^{v}\frac{1}{c^{2}}n+\Lambda_{\alpha }^{v}\frac{1}{c^{4}}\left(nv_{\mu }v^{\alpha }+ne\delta_{\mu }^{\alpha }-t_{\mu }^{\alpha }\right)+ \end{array}$$

(49) $$\begin{array}{ccc} & \;+ & \frac{1}{c^{3}}\Lambda^{n}\left(1-\frac{v^{2}}{c^{2}}\right)v_{\mu }+\frac{1}{c^{5}}\left(\Phi_{\mu }+\Lambda^{e}\left(2q_{\mu }+v_{\nu }t_{\mu }^{\nu }\right)\right) \end{array}$$

(50) $$\begin{array}{ccc} \;O(\frac{1}{c^{2}}) & : & \Lambda_{\alpha }^{v}=0 \end{array}$$

(51) $$\begin{array}{ccc} O(\frac{1}{c^{3}}) & : & \Lambda_{\alpha }^{v}=\frac{1}{c}\Lambda^{n}\frac{1}{n}v_{\alpha }=-\frac{1}{c^{3}}\frac{\partial \eta }{\partial n}v_{\alpha }\\ \partial_{\gamma }\partial_{\mu }z & : & 0=-\frac{1}{c^{2}}\Lambda_{\beta }^{v}\left(\frac{\partial t^{\beta \gamma }}{\partial \partial_{\mu }z}-\frac{1}{c^{2}}v^{\gamma }v_{\alpha }\frac{\partial t^{\beta \alpha }}{\partial \partial_{\mu }z}-\frac{v^{2}}{c^{2}}\frac{\partial t^{\beta \gamma }}{\partial \partial_{\mu }z}\right)+\\ & + & \frac{1}{c^{3}}\left(\frac{\partial \Phi^{\gamma }}{\partial \partial_{\mu }z}+\Lambda^{e}\frac{\partial q^{\gamma }}{\partial \partial_{\mu }z}\right)+ \end{array}$$

(52) $$\begin{array}{ccc} & \;+ & \frac{1}{c^{5}}\left(-v^{\gamma }v_{\alpha }\frac{\partial \Phi^{\alpha }}{\partial \partial_{\mu }z}-\Lambda^{e}v^{\gamma }v_{\alpha }\frac{\partial q^{\alpha }}{\partial \partial_{\mu }z}-\Lambda^{e}\frac{v^{2}}{2}\frac{\partial q^{\gamma }}{\partial \partial_{\mu }z}\right) \end{array}$$

(53) $$\begin{array}{ccc} \;O(\frac{1}{c^{3}}) & : & \frac{1}{c^{3}}\left(\frac{\partial \Phi^{\gamma }}{\partial \partial_{\mu }z}+\Lambda^{e}\frac{\partial q^{\gamma }}{\partial \partial_{\mu }z}\right)=0, \end{array}$$

where *z* is the abbreviation for the variables $z\in \{n,e,v^{\epsilon }\}$. In the equations of lowest order in $\frac{1}{c}$, i.e., keeping the terms up to order $\frac{1}{c^{3}}$, it has been made use of the fact that $\Lambda^{v}$ is of the order $\frac{1}{c^{2}}$ and, therefore, leads to higher order correction terms.

In the residual inequality, the terms without higher derivatives are combined:

(54) $$\begin{array}{c} \sigma =\frac{\partial \Phi^{\alpha }}{\partial n}\partial_{\alpha }n+\frac{\partial \Phi^{\alpha }}{\partial e}\partial_{\alpha }e+\\ +\frac{1}{c^{2}}\left(-v^{\mu }\partial_{\mu }v_{\alpha }\Phi^{\alpha }-v^{\mu }v_{\alpha }\left(\frac{\partial \Phi^{\alpha }}{\partial n}\partial_{\mu }n+\frac{\partial \Phi^{\alpha }}{\partial e}\partial_{\mu }e\right)\right)+c^{2}\Lambda^{n}n\partial_{\mu }v^{\mu }+\\ +\Lambda^{e}\left(\left(1-\frac{1}{2}\frac{v^{2}}{c^{2}}\right)\left(\frac{\partial q^{\mu }}{\partial n}\partial_{\mu }n+\frac{\partial q^{\mu }}{\partial e}\partial_{\mu }e\right)-t^{\mu \nu }\partial_{\mu }v_{\nu }\right)+\\ +\Lambda^{e}\frac{1}{c^{2}}\left(-v_{\mu }v^{\alpha }\left(\frac{\partial q^{\mu }}{\partial n}\partial_{\alpha }n+\frac{\partial q^{\mu }}{\partial e}\partial_{\alpha }e\right)-q^{\mu }v_{\nu }\partial_{\mu }v^{\nu }+t^{\mu \nu }v_{\mu }v^{\alpha }\partial_{\alpha }v_{\nu }\right)+\\ +\Lambda_{\mu }^{v}c\left(-\frac{\partial t^{\mu \nu }}{\partial n}\partial_{\nu }n-\frac{\partial t^{\mu \nu }}{\partial e}\partial_{\nu }e\right)+\Lambda_{\mu }^{v}\frac{1}{c}\left(v^{2}\left(\frac{\partial t^{\mu \nu }}{\partial n}\partial_{\nu }n+\frac{\partial t^{\mu \nu }}{\partial e}\partial_{\nu }e\right)+\right.\\ +v_{\nu }v^{\alpha }\left(\frac{\partial t^{\mu \nu }}{\partial n}\partial_{\alpha }n+\frac{\partial t^{\mu \nu }}{\partial e}\partial_{\alpha }e\right)+v^{\alpha }\partial_{\alpha }v_{\nu }t^{\mu \nu }-\\ -\left.v^{\mu }t^{\alpha \nu }\partial_{\alpha }v_{\nu }+q_{\nu }\partial^{\mu }v^{\nu }+q^{\mu }\partial_{\nu }v^{\nu }\frac{}{}\right) \end{array}$$

### 4.1. Results of the Exploitation of the Dissipation Inequality up to Order $\frac{1}{c^{3}}$

The results of the Liu procedure in the lowest non-trivial order are presented after each of the Liu equations. Equations (44), (46) and (48) show that in this approximation the entropy does not depend on the gradients, i.e., η(n,e) as in the non-relativisitic theory. Consequently, $\Lambda^{e}$ and $\Lambda^{n}$ also do not depend on the gradients. Together with the fact, that $\frac{1}{c^{2}}\Lambda^{v}$ is a higher order correction term, Equation (52) result in

(55) $$\begin{array}{c} \frac{\partial (\Phi^{\gamma }+\Lambda^{e}q^{\gamma })}{\partial \partial_{\mu }z}=0. \end{array}$$

As in the non-relativistic theory, the extra entropy flux is a function of the scalar equilibrium variables only: k(n,e), and consequently k=0. The constitutive equation for the entropy flux $\Phi =\frac{q}{T}$ is the classical one of the Thermodynamics of Irreversible Processes.

The entropy production (54) reduces to

(56) $$\begin{array}{c} \sigma =\frac{\partial \Phi^{\alpha }}{\partial n}\partial_{\alpha }n+\frac{\partial \Phi^{\alpha }}{\partial e}\partial_{\alpha }e+c^{2}\Lambda^{n}n\partial_{\mu }v^{\mu }+\\ +\Lambda^{e}\left(\frac{\partial q^{\mu }}{\partial n}\partial_{\mu }n+\frac{\partial q^{\mu }}{\partial e}\partial_{\mu }e-t^{\mu \nu }\partial_{\mu }v_{\nu }\right) \end{array}$$

Because $\Phi +\Lambda^{e}q$ is a function of *e* and *n* only in this approximation,

(57) $$\begin{array}{c} \frac{\partial \Phi^{\alpha }}{\partial n}\partial_{\alpha }n+\frac{\partial \Phi^{\alpha }}{\partial e}\partial_{\alpha }e+\Lambda^{e}\left(\frac{\partial q^{\mu }}{\partial n}\partial_{\mu }n+\frac{\partial q^{\mu }}{\partial e}\partial_{\mu }e\right)\\ =\partial_{\alpha }\left(\Phi^{\alpha }+\Lambda^{e}q^{\alpha }\right)-q^{\alpha }\partial_{\alpha }\Lambda^{e}=-q^{\alpha }\partial_{\alpha }\Lambda^{e}=q^{\alpha }\partial_{\alpha }\left(n\frac{\partial \eta }{\partial e}\right). \end{array}$$

The entropy production can be rewritten as

(58) $$\begin{array}{c} \sigma =q^{\alpha }\partial_{\alpha }\left(n\frac{\partial \eta }{\partial e}\right)+n\frac{\partial \eta }{\partial e}t^{\mu \nu }\partial_{\mu }v_{\nu }-n^{2}\frac{\partial \eta }{\partial n}\partial_{\mu }v^{\mu } \end{array}$$

Identifying $n\frac{\partial \eta }{\partial e}$ with $\frac{1}{T}$ as in equilibrium, the entropy production has the classical form as in Irreversible Thermodynamics

(59) $$\begin{array}{c} \sigma =-\frac{1}{T^{2}}q^{\alpha }\partial_{\alpha }T+\frac{1}{T}t^{\mu \nu }\partial_{\mu }v_{\nu }-n^{2}\frac{\partial \eta }{\partial n}\partial_{\mu }v^{\mu } \end{array}$$

### 4.2. Results of the Exploitation of the Dissipation Inequality up to Order $\frac{1}{c^{5}}$

#### 4.2.1. Restrictions on Constitutive Functions

In Equations (43), (45) and (47), the multipliers $\Lambda^{e}$ and $\Lambda^{v}$ are inserted and terms of order higher than $\frac{1}{c^{5}}$ are neglected. Due to Equation (51), $\Lambda^{v}$ is of the order $\frac{1}{c^{3}}$, and the terms $\frac{1}{c^{4}}\Lambda_{\alpha }^{v}\left(\ldots \right)$ in the Liu Equations (43)–(47), are higher order correction terms, neglected in this approximation.

The result is the following restrictions

(60) $$\begin{array}{c} 0=\frac{1}{c^{3}}n\frac{\partial \eta }{\partial \partial_{\mu }z}\left(1-\frac{v^{2}}{c^{2}}\right)+\frac{1}{c^{5}}v_{\alpha }\left(\frac{\partial \Phi^{\alpha }}{\partial \partial_{\mu }z}-n\frac{\partial \eta }{\partial e}\frac{\partial q^{\alpha }}{\partial \partial_{\mu }z}\right) \end{array}$$

or

(61) $$\begin{array}{c} \frac{1}{c^{2}}v_{\alpha }\frac{\partial k^{\alpha }}{\partial \partial_{\mu }z}=-n\frac{\partial \eta }{\partial \partial_{\mu }z}\left(1-\frac{v^{2}}{c^{2}}\right)-\frac{1}{c^{2}}v_{\alpha }q^{\alpha }n\frac{\partial^{2}\eta }{\partial e\partial \partial_{\mu }z} \end{array}$$

with $z\in \{n,e,v^{\epsilon }\}$ and $k=\Phi +\Lambda^{e}q$. This extra entropy flux is not necessarily zero, and the constitutive law of relativistic Irreversible Thermodynamics is an assumption. On the other hand, if $\frac{\partial \eta }{\partial \partial_{\mu }z}=0$, i.e., the entropy depends on the equilibrium variables only, then the right hand side of Equation (61) is zero, and the extra entropy flux vanishes. In this order of approximation, the constitutive equation for the entropy flux as heat flux divided by temperature is a consequence of the local equilibrium assumption for the entropy density.

Inserting the multipliers in Equation (52) and neglecting terms of a higher order than $\frac{1}{c^{5}}$ leads to the restrictions

(62) $$\begin{array}{c} \frac{1}{c^{5}}\frac{1}{n}\frac{\partial \eta }{\partial n}v_{\beta }\frac{\partial t^{\beta \gamma }}{\partial \partial_{\mu }z}+\frac{1}{c^{3}}\left(\frac{\partial \Phi^{\gamma }}{\partial \partial_{\mu }z}+\Lambda^{e}\frac{\partial q^{\gamma }}{\partial \partial_{\mu }z}\right)+\\ +\frac{1}{c^{5}}\left(-v^{\gamma }v_{\alpha }\frac{\partial \Phi^{\alpha }}{\partial \partial_{\mu }z}+n\frac{\partial \eta }{\partial e}v\gamma v_{\alpha }\frac{\partial q^{\alpha }}{\partial \partial_{\mu }z}+n\frac{\partial \eta }{\partial e}\frac{v^{2}}{2}\frac{\partial q^{\gamma }}{\partial \partial_{\mu }z}\right)=0. \end{array}$$

In the special case that $\Phi +\Lambda^{e}q=0$, this reduces to

(63) $$\begin{array}{c} \frac{\partial \eta }{\partial n}v_{\beta }\frac{\partial t^{\beta \gamma }}{\partial \partial_{\mu }z}+n^{2}\frac{\partial \eta }{\partial e}\frac{v^{2}}{2}\frac{\partial q^{\gamma }}{\partial \partial_{\mu }z}=0, \end{array}$$

$z\in \{n,e,v^{\epsilon }\}$. The gradient-dependent stress tensor and heat flux are not independent of each other but are related by Equation (63).

#### 4.2.2. Entropy Production

Because $\Lambda_{\alpha }^{v}=-\frac{1}{c^{3}}\frac{1}{n}\frac{\partial \eta }{\partial n}v_{\alpha }$, terms with the factor $\frac{1}{c}\Lambda_{\mu }^{v}$ are higher order terms and are neglected.

(64) $$\begin{array}{c} \sigma =\frac{\partial \Phi^{\alpha }}{\partial n}\partial_{\alpha }n+\frac{\partial \Phi^{\alpha }}{\partial e}\partial_{\alpha }e+\\ \frac{1}{c^{2}}\left(-v^{\mu }\partial_{\mu }v_{\alpha }\Phi^{\alpha }-v^{\mu }v_{\alpha }\left(\frac{\partial \Phi^{\alpha }}{\partial n}\partial_{\mu }n+\frac{\partial \Phi^{\alpha }}{\partial e}\partial_{\mu }e\right)\right)+c^{2}\Lambda^{n}n\partial_{\mu }v^{\mu }\\ +\Lambda^{e}\left(\left(1-\frac{1}{2}\frac{v^{2}}{c^{2}}\right)\left(\frac{\partial q^{\mu }}{\partial n}\partial_{\mu }n+\frac{\partial q^{\mu }}{\partial e}\partial_{\mu }e\right)-t^{\mu \nu }\partial_{\mu }v_{\nu }\right)\\ +\Lambda^{e}\frac{1}{c^{2}}\left(-v_{\mu }v^{\alpha }\left(\frac{\partial q^{\mu }}{\partial n}\partial_{\alpha }n+\frac{\partial q^{\mu }}{\partial e}\partial_{\alpha }e\right)-q^{\mu }v^{\nu }\partial_{\mu }v_{\nu }+t^{\mu \nu }v_{\mu }v^{\alpha }\partial_{\alpha }v_{\nu }\right)+\\ +\Lambda_{\mu }^{v}c\left(-\frac{\partial t^{\mu \nu }}{\partial n}\partial_{\nu }n-\frac{\partial t^{\mu \nu }}{\partial e}\partial_{\nu }e\right) \end{array}$$

Equation (52)$\cdot c^{3}$ is added to Equation (64). After some rearrangement, this results in the following expression for the entropy production in three-dimensional notation

(65) $$\begin{array}{c} \sigma =\underset{relativistic\;correction\;to\;the\;entropy\;flux}{\underset{︸}{\nabla \cdot k^{rel}}}\\ \underset{heat\;conduction}{\underset{︸}{-q\cdot \nabla \Lambda^{e}}}+\underset{volume\;viscosity}{\underset{︸}{\left(\nabla \cdot v\right)\left(-\frac{\partial \eta }{\partial n}+\frac{1}{c^{2}}v\cdot \left(\Phi +\Lambda^{e}q\right)\right)}}\\ -\Lambda^{e}\underset{shear\;viscosity}{\underset{︸}{\left(\delta -\frac{vv}{c^{2}}\right):\left(t\cdot \nabla v\right)}}-\underset{purely\;relativistic\;correction\;terms}{\underset{︸}{\frac{v^{2}}{2c^{2}}\Lambda^{e}\nabla \cdot q+\frac{1}{c^{2}}\frac{1}{n}\frac{\partial \eta }{\partial n}v\cdot \left(\nabla \cdot t\right)}}, \end{array}$$

where we introduced the abbreviation

(66) $$k^{rel}=\left(\delta -\frac{vv}{c^{2}}\right)\cdot \left(\Phi +\Lambda^{e}q\right).$$

The volume viscosity term defines the relativistic dynamic pressure

(67) $$-\frac{\partial \eta }{\partial n}+\frac{1}{c^{2}}v\cdot \left(\Phi +\Lambda^{e}q\right)-trace\left(t\right)\frac{\Lambda^{e}}{3}=p_{dyn}^{rel}.$$

In the **special case of local equilibrium**, the specific entropy depends on the equilibrium variables only η(n,e). In this case Equation (60) reduces to

(68) $$\begin{array}{c} v_{\alpha }\left(\frac{\partial \Phi^{\alpha }}{\partial \partial_{\mu }z}-n\frac{\partial \eta }{\partial e}\frac{\partial q^{\alpha }}{\partial \partial_{\mu }z}\right)=\frac{\partial }{\partial \partial_{\mu }z}\left(v_{\alpha }\left(\Phi^{\alpha }-n\frac{\partial \eta }{\partial e}q^{\alpha }\right)\right)=0, \end{array}$$

i.e., $v_{\alpha }\left(\Phi^{\alpha }-n\frac{\partial \eta }{\partial e}q^{\alpha }\right)\left(e,n\right)$ is a function of the equilibrium variables. Because ***v*** is independent of *e* and *n*, the vector $\Phi -n\frac{\partial \eta }{\partial e}q$ is zero, and the entropy flux is the classical one of TIP.

In this special case, the entropy production reduces to

(69) $$\begin{array}{c} \sigma =-\frac{v^{2}}{2c^{2}}\nabla \cdot \left(\Lambda^{e}q\right)-\left(1-\frac{v^{2}}{2c^{2}}\right)q\cdot \nabla \Lambda^{e}-\left(\nabla \cdot v\right)\frac{\partial \eta }{\partial n}+\\ \Lambda^{e}\left(\delta -\frac{vv}{c^{2}}\right):\left(t\cdot \nabla v\right)+\frac{1}{c^{2}}\frac{1}{n}\frac{\partial \eta }{\partial n}v\cdot \left(\nabla \cdot t\right), \end{array}$$

The first term is the relativistic correction term of the entropy flux. The contributions to the entropy production of heat flux and viscous flow are of the classical form of TIP with relativistic corrections as prefactors. The last term is a relativistic correction term, also related to viscous flow.

## 5. Conclusions

The restrictions on constitutive functions by the Second Law of Thermodynamics have been exploited by the method of Liu, taking into account the relativistic balance equations for an observer up to the order of $\frac{1}{c^{5}}$. To the lowest non-trivial order of $\frac{1}{c^{3}}$, the results of the non-relativistic exploitation of the entropy inequality are recovered. These are the assumptions of TIP—local equilibrium and entropy flux as heat flux divided by temperature—as well the same expression for the entropy production as in TIP. In the next order $\frac{1}{c^{5}}$, an extra entropy flux $\Phi -\frac{q}{T}$ is not necessarily zero and the entropy may depend on gradients. Here, the assumptions of TIP cannot be derived from the more general method of Liu. However, if the entropy density is local, i.e., depends on particle number and internal energy only, it follows that $\Phi =\frac{q}{T}$. Even in this case, there appears to be a relativistic correction term in the entropy production, which is not of the form of the classical TIP.

## Data Availability

No new data were created or analyzed in this study. Data sharing is not applicable to this article.
